# An Electron paramagnetic resonance (EPR) spin labeling study in HT-29 Colon adenocarcinoma cells after Hypericin-mediated photodynamic therapy

**DOI:** 10.1186/s12860-019-0205-4

**Published:** 2019-06-20

**Authors:** D. Yonar, A. Kılıç Süloğlu, G. Selmanoğlu, M. M. Sünnetçioğlu

**Affiliations:** 1 Department of Physics Engineering, Faculty of Engineering, Hacettepe University, Beytepe, 06800 Ankara, Turkey; 2 Department of Biology, Faculty of Science, Hacettepe University, Beytepe, Ankara, Turkey; 3 Present address: Department of Biophysics, Faculty of Medicine, Yuksek Ihtisas University, Ankara, Turkey

**Keywords:** Photodynamic therapy, Hypericin, Membrane fluidity, EPR spin labeling

## Abstract

**Background:**

Colon cancer affects 1.23 million people worldwide and is the third most common malignant disease in men and the second in women. The only curative treatment is surgical resection, but a significant number of patients develop local recurrence or distant metastases. One of the alternative treatment methods for colon cancer is photodynamic therapy (PDT). In recent years, hypericin (HYP) derived from *Hypericum perforatum* has been suggested as a strong candidate photosensitizer for PDT. Our interest is focused on the biophysical changes in colon cancer cells in relation to HYP-mediated PDT.

**Results:**

In this study, HYP-mediated PDT at 0.04, 0.08 or 0.15 μM HYP concentrations was performed in HT-29 colon adenocarcinoma cells and the Electron Paramagnetic Resonance (EPR) spectra of the spin labeled cells were obtained. Plasma membranes are already heterogeneous structures; the presence of cancer cells increased the heterogeneity and also fluidity of the plasma membranes. Therefore, the obtained spectra were evaluated by EPRSIMC program, which provides the calculation of heterogeneous structures up to four spectral components with different fluidity characteristics. Generally, two motional patterns were obtained from calculations and the number of them increased at the highest concentration. As the order parameters of the most populated components compared, an increase was observed depending on the HYP concentration. However, because of the heterogeneous structure of membrane, the order parameters of the less populated components did not exhibit a regular distribution.

**Conclusion:**

After HYP-mediated PDT, concentration dependent changes were observed in the domain parameters indicating an increase in the HYP accumulation.

## Background

CRC (colorectal cancer) is the third most common type of cancer that causes morbidity and mortality worldwide, with 1.65 million new cases and almost 835,000 deaths in 2015 [1]. Approximately 70% of these cases arise in the colon. Treatments used for colorectal cancer may include some combinations of surgery radiation therapy, chemotherapy and targeted therapy. Early diagnosis and efficient treatment strategies are substantial for patient survival. One of the new and eminent candidates for cancer treatment is photodynamic therapy (PDT) that uses a photosensitizer (PS) and a particular type of light [2].

**Fig. 1:**
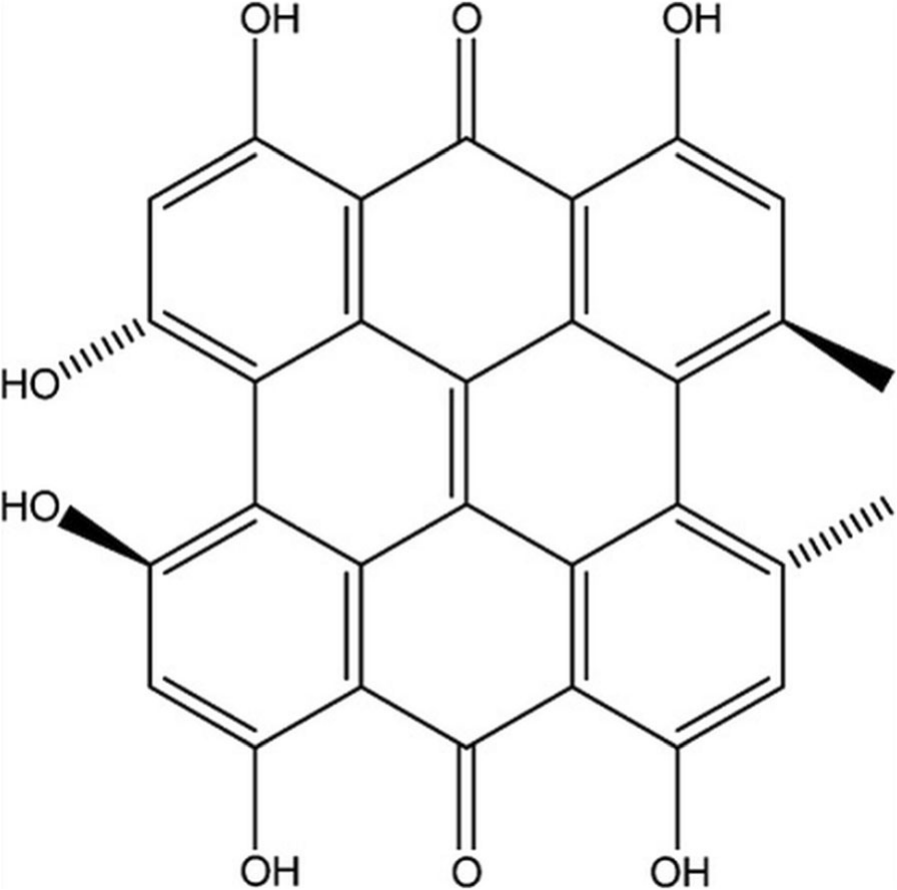
Chemical structure of hypericin

Hypericin (HYP), a natural photosensitizer extracted from *Hypericum perforatum* (commonly known as *St. John’s Wort*), is a highly hydrophobic compound (Fig. 1). HYP’s hydrophobic character makes it insoluble in water and non-polar solvents, where it forms non-fluorescent aggregates. These aggregates significantly suppress its photodynamic activity. On the contrary, it is well dissolved in polar organic substances like dimethyl sulfoxide (DMSO) and in this case, HYP possesses a strong fluorescence and high quantum yield of singlet oxygen formation. Because of its photo-induced cytotoxicity and selective antitumor features, HYP has been recently gained importance in PDT of cancer [3–5]. HYP can freely diffuse into tumor cells or it incorporates into cells via endocytosis. Intracellularly it localizes in various cellular or subcellular regions such as plasma membrane, endoplasmic reticulum, Golgi apparatus, lysosomes and mitochondria. Recent studies have showed the HYP accumulation in the perinuclear region of the tumor cells [6, 7]. It has been suggested that after treatment of HYP-mediated PDT, cell death pathways induced depending on the concentration of treated HYP. Low-dose concentrations of HYP induce growth stimulation via the p38 or JNK survival pathways. Unlike the effect of a low dose, a high dose of HYP leads to activation of stress-response pathways that trigger apoptosis or autophagic cell death. If the damage is too severe, the cell dies necrotically [8].

Dysadherin, which is a cell membrane glycoprotein and anti-adhesion molecule was investigated previously by Kılıç Süloğlu et al. [9]. The authors showed that HYP-mediated PDT decreased the dysadherin expression and F-actin organization both in HT-29 and Caco-2 colon cancer cells. Lactate dehydrogenase (LDH) is a cytoplasmic enzyme, which is a good biomarker of damaged cell membrane. In another study with HT-29 cells, HYP-mediated PDT caused an increased LDH leakage, and alterations in cell cycle progression indicating the dead cells [10]. These membrane-related changes due to the PDT by HYP encourage us to investigate detailed analysis of cell membrane alterations by EPR spin labeling method.

EPR technique is a valuable tool based on the detection of the free radicals within the sample. In case the number of free radicals in the sample is not sufficient, it is possible to investigate the sample with the use of spin labels, which are stable free radicals and when they are inserted into the sample, the information about the environment of the spin label is obtained precisely from the recorded EPR spectra. Using this technique, various studies were performed on cancer research [11–13]. These studies indicated that the alterations in the lipid composition might be correlated with the development of some cancer types. It is possible to follow therapeutic changes in the tumor cells by incorporation of drugs via EPR technique. In the present study, it is aimed to observe the effects of HYP-mediated PDT in in vitro models of the colon cancer cells. For that purpose, colon cancer cell line (HT-29) was treated with different concentrations of HYP, while the non-HYP treated group was used as control. Using EPR spin labeling technique, we investigated the alterations caused by HYP-mediated PDT on lipid dynamics in the tumor cell system for the first time. EPR spectroscopy provides a sensitive way of determining dynamic parameters of lipid system by using a spin label that positions itself into the lipid bilayer [13, 14].

## Methods

### Cell culture

HT-29 cells were obtained from HUKUK, Foot and Mouth Disease Institute, Ankara, Turkey. The cells were routinely maintained in Dulbeccos MEM containing 10% fetal bovine serum at 37 °C (310 K) in a controlled atmosphere of 5% CO_2_ and 90% relative humidity. The cells were seeded in 25 cm^2^ flask (60 × 10^4^) then cultivated for 24 h. Hypericin (HYP) HPLC grade (AppliChem, Germany), was prepared as a stock solution in DMSO and then diluted to studied concentrations. The cells were treated with different concentrations of HYP (0.04, 0.08, 0.15 and 0.40 μM) in dark conditions for 24 h and irradiated with a device consisting twelve L18 W/830 fluorescent tubes (Osram, Germany) with maximum emission in the range of λ = 530–620 nm which covers the maximum absorbance of HYP. The total light dose was 4 J/cm^2^.

### EPR measurements

Spin labelling of the cells was performed using the 16-doxyl stearic acid (16-DS) spin label. 24 h after irradiation HT29 cells incubated with 10^− 3^ M 16-DS spin label suspension for 60 min at 37 °C. The cells were spin labelled by incubating a suspension of cells to a final concentration of 10^− 4^ M 16-DS. Unbound spin labels were removed by washing the cells in PBS and centrifugated at 1200 *g* for 4 min until no free spin label signal was observed in the supernatant. The pellet of the cells was transferred to a disposable glass capillary. EPR measurements were performed on a X-band Bruker EMX-131 spectrometer (Bruker BioSpin GmbH, Rheinstetten, Germany) with ER4103TM cylindirical cavity. The following spectral conditions were used: modulation frequency 100 kHz, microwave power 10 mW and modulation amplitude 0.5 mT at 298 and 310 K. Sample temperature was controlled to ±1 K by Bruker VT4111 temperature controller. EPR measurements of each sample were measured at least two times. After approximately 4 h later from the first measurements, a decrease in EPR peak heights was observed which indicates a part of spin label becomes EPR silent in time.

### Determination of empirical correlation time τ_emp_

The EPR spectral line shape of the spin label describes the properties of its surroundings. Rotational correlation time, the time required for the reorientation of the spin label within the membrane, depends on the microviscosity of the environment and increase in the microviscosity increases it.

**Fig. 2:**
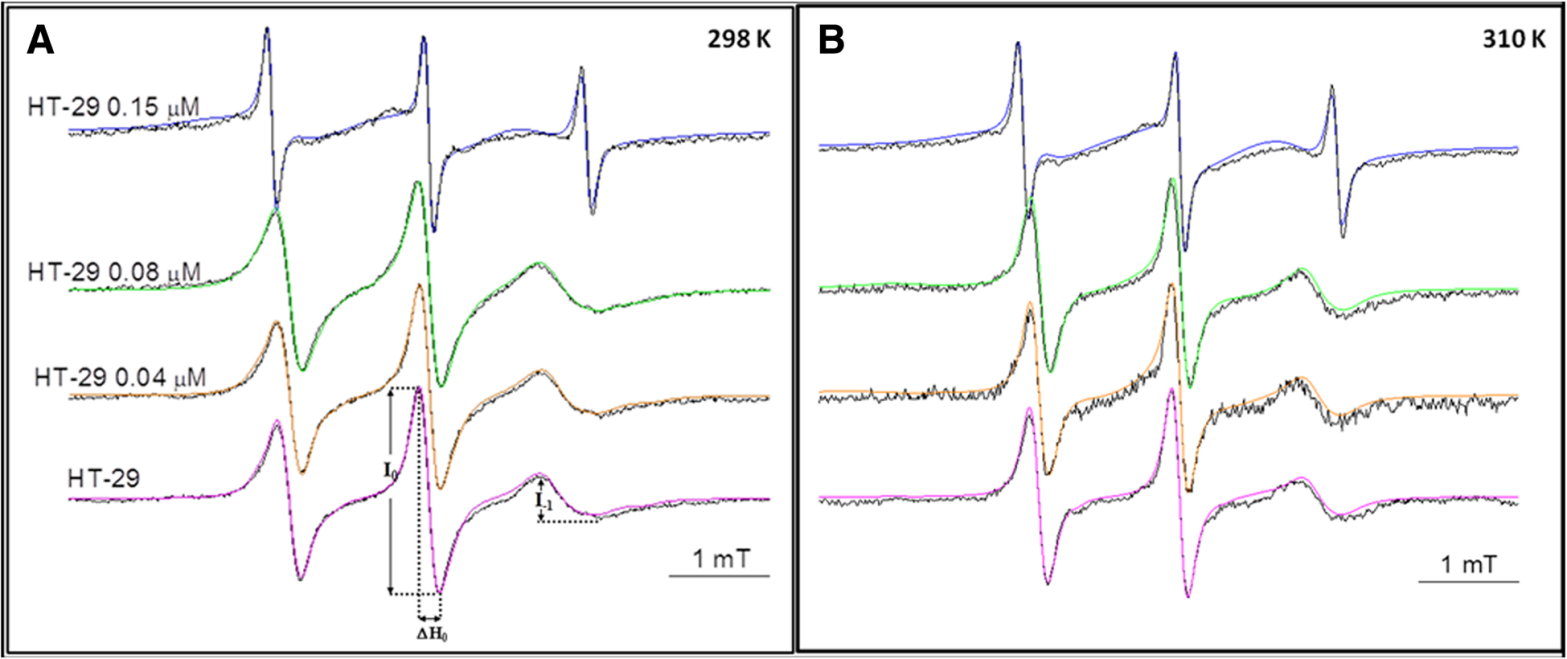
Experimental (black lines) and calculated spectra (colored lines) of 16-DS spin labeled control and HYP mediated HT-29 cells at **a**) 298 K, **b**) 310 K

For a rough estimation of membrane fluidity differences between different cell lines empirical correlation time τ_emp_ was calculated as given below.

1 $$ {\tau}_{emp}=K\Delta {B}_0\left({\left(\frac{I_0}{I_{-1}}\right)}^{1/2}-1\right) $$

where I_− 1_ and I_0_ are high and middle field intensities of the EPR spectra, ΔB_0_ is the line width of the middle field line (measured as shown in Fig. 2), and K is a constant typical for the spin label [15].

### Computer simulation of EPR spectra

Simulations of the spectra were also performed to get more precise description of membrane characteristics. Since cell plasma membranes are heterogeneous, this heterogeneity is reflected to EPR spectra in the form of several spectral components (domains) having different values of physical parameters. These domains reflect different modes of restricted rotational motion of the spin probe molecules in different membrane environments. Therefore, the EPR spectrum is composed of several spectral components and each component is described with a set of spectral parameters [16]. EPRSIM-C simulations provide four domains simulations and so that the number of the domains and their physical parameters were determined from the simulations of recorded spectra. The EPRSIM-C library includes several algorithms for the simulation models. In the present study, *Anisotropic Tumbling with Partial Averaging of All Rotations (MES)* model was used to simulate EPR spectra of nitroxide spin probes in an anisotropic environment like a membrane. This model includes two cone angle parameters (the main cone and asymmetry angles, θ, φ, respectively) describing the wobble model to generalize restriction of the rotational motion. Since multiple optimization routines provide a huge number of data sets, most significant and reliable solutions have to be found. This was done by the GHOST condensation algorithm. The groups of solutions are represented by two-dimensional cross-section diagrams such as *S–* τ_c_, *S–W*, *S–p*a, or θ–φ, θ–τ_c_, θ–W, θ–*p*a (*S*, τ_c_, *W*, and *p*a are typical model parameters: order parameter, effective rotational correlation time, additional broadening constant, and polarity correction factor on the hyperfine tensor) [17]. However, the differences in the EPR spectra depend mostly on the order parameter and relative proportions between the spectral components. Order parameter (S) is related to time averaged amplitude of rotational motion of nitroxide group relative to the normal of the membrane surface (S = 1 for perfectly oriented molecules and S = 0 for isotropic motion of molecules).

### Statistics

Statistical data analyses were carried out using the GraphPad Prism 6 software package. Statistical comparisons for in vitro data were evaluated by using one-way analysis of variance (one-way ANOVA) and Dunnett’s multiple comparison tests. Differences were considered to be statistically significant at the level of *p* < 0.05. The results were represented as the mean values of parameters together with the S.E.M.

## Results and discussion

In the present study, the effects of HYP-mediated PDT in in vitro models of the colon cancer cells (HT-29) were investigated by using 16-DS spin label, which has the nitroxide moiety located at the end of the methylene chain. Experimental and calculated spectra of HT-29 colon cancer cells and HYP-mediated ones were given at 298 K and 310 K in Fig. 2. The calculated spectra are the superimposition of all the domains spectra with different parameters.

**Fig. 3:**
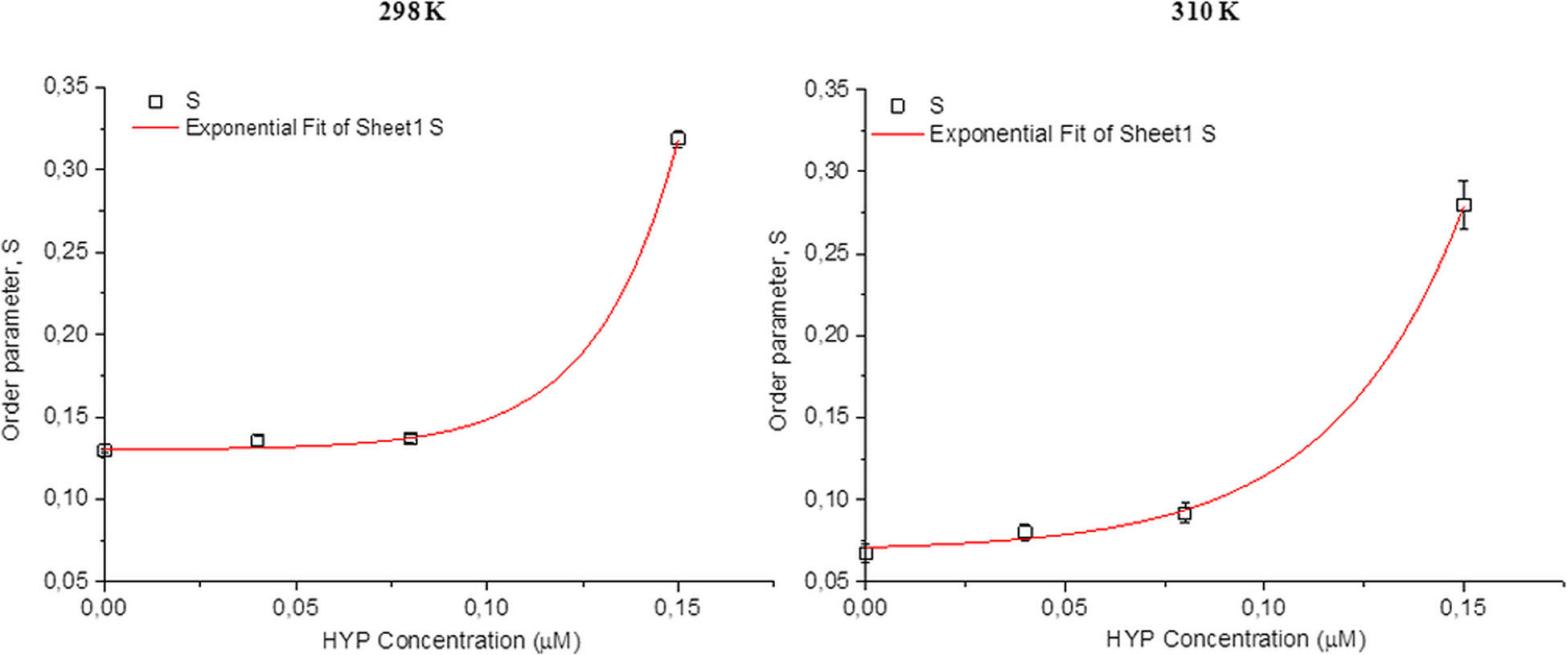
Concentration dependent change in the order parameter of HT-29 colon cancer cells at 298 and 310 K

**Fig. 4:**
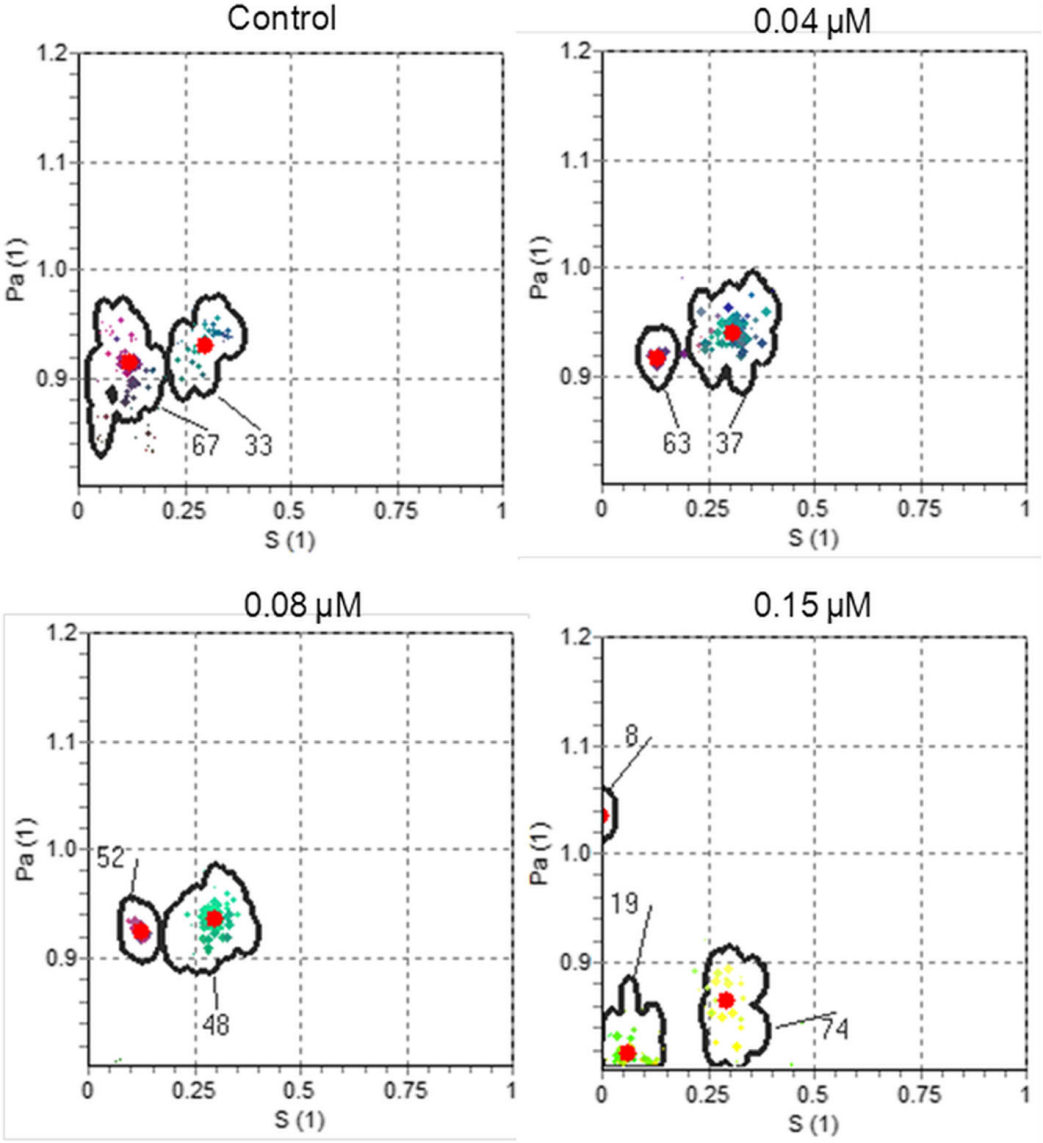
Examples of the GHOST diagrams, defined by the RGB specification where the intensity of each color component (red, green, and blue, respectively) represents the relative value of the spectral parameters (τ_c_, W, pa), for control and HYP treated HT-29 cells at 298 K. The red points show the weighted mean and peripheral colored points show the distribution of solutions

The obtained spectra were simulated by EPRSIM-C program, which provides the calculation of heterogeneous structures up to four spectral components with different fluidity characteristics. Hence, the information on domain (spectral components with different motional pattern) properties of cells and the physical parameters of these components were acquired. The ordering and dynamics of the cellular membrane are described with the order parameter, S and rotational correlation time, τ_c_. The order parameters and rotational correlation times for non-treated and treated cells were obtained from simulation program. However, the empirical correlation time (τ_emp_) was determined from the spectra as seen from Fig. 2 by Eq. 1. The observed changes in the order parameters are represented in Fig. 3. As the order parameters of the most populated components compared, an increase was observed with the increased HYP concentrations at 298 K and 310 K. The order parameters calculated from the weighted mean of the domains exhibited a very similar behavior with an increasing concentration. In fact, the observed increase is the result of the change in the population of the domains. The population of the most ordered domain ascends with the increasing concentration of HYP (Fig. 4). The increase in the order parameters up to 0.15 μM HYP concentrations for both temperatures was linear, whereas the dramatic increase was observed at 0.15 μM HYP concentration*.* To justify this result, the calculated order parameter for 0.15 μM HYP concentration was obtained from the weighted mean of all domains and similar results were achieved.

As clearly seen from Fig. 2, the shape of the spectrum at 0.15 μM is different from lower HYP concentrations for both 298 K and 310 K. An increase in the number of domains and a change in domain parameters were observed for 0.15 μM HYP mediated samples. There exists a broad spectrum under the isotropic part. This broad part includes some contribution from spin-spin exchange interaction because of close neighborhood of the nitroxide spin labels. The reason of this interaction is higher HYP accumulation in HT-29 cells in the 0.15 μM HYP treatment group. However, it was possible to simulate 0.15 μM HYP mediated samples using MES. In another study, which is aimed to clarify the effect of HYP-mediated PDT on colon cancer cells, the relative intracellular HYP accumulation was evaluated by measuring fluorescence intensity and the increase in intracellular HYP accumulation. The observed cellular HYP uptake was high in Caco-2 cells for 0.04 and 0.08 μM HYP concentrations but, in HT-29 cells HYP accumulation was higher in the 0.15 μM HYP treatment group [9]. In HT-29 cells, a dose dependent HYP accumulation and increase in apoptotic index was found and both were higher in the 0.15 μM HYP treatment group. The obtained EPR results justified the information about intracellular HYP accumulation. The preferential sub-cellular location of free and liposome encapsulated HYP within cells is important for HYP-PDT studies. Recent studies showed the HYP accumulation in the perinuclear region of the tumor cells [6, 7].

At 298 K, as clearly seen from GHOST diagrams (*S–p*a), the order parameter of the most populated domain regularly ascended with the increasing HYP concentration (Fig. 4).

Rotational correlation times provide information about cell fluidity changes. Membrane fluidity depends on the molecular composition of the domains, especially on lipid and protein composition as well as on the ratio between cholesterol and other lipids. Alterations in membrane fluidity have been involved in several fundamental cellular functions. It has been shown that an increase in cell membrane fluidity is significantly correlated with malignant potential of the tumor cells [11, 13, 18].

**Fig. 5:**
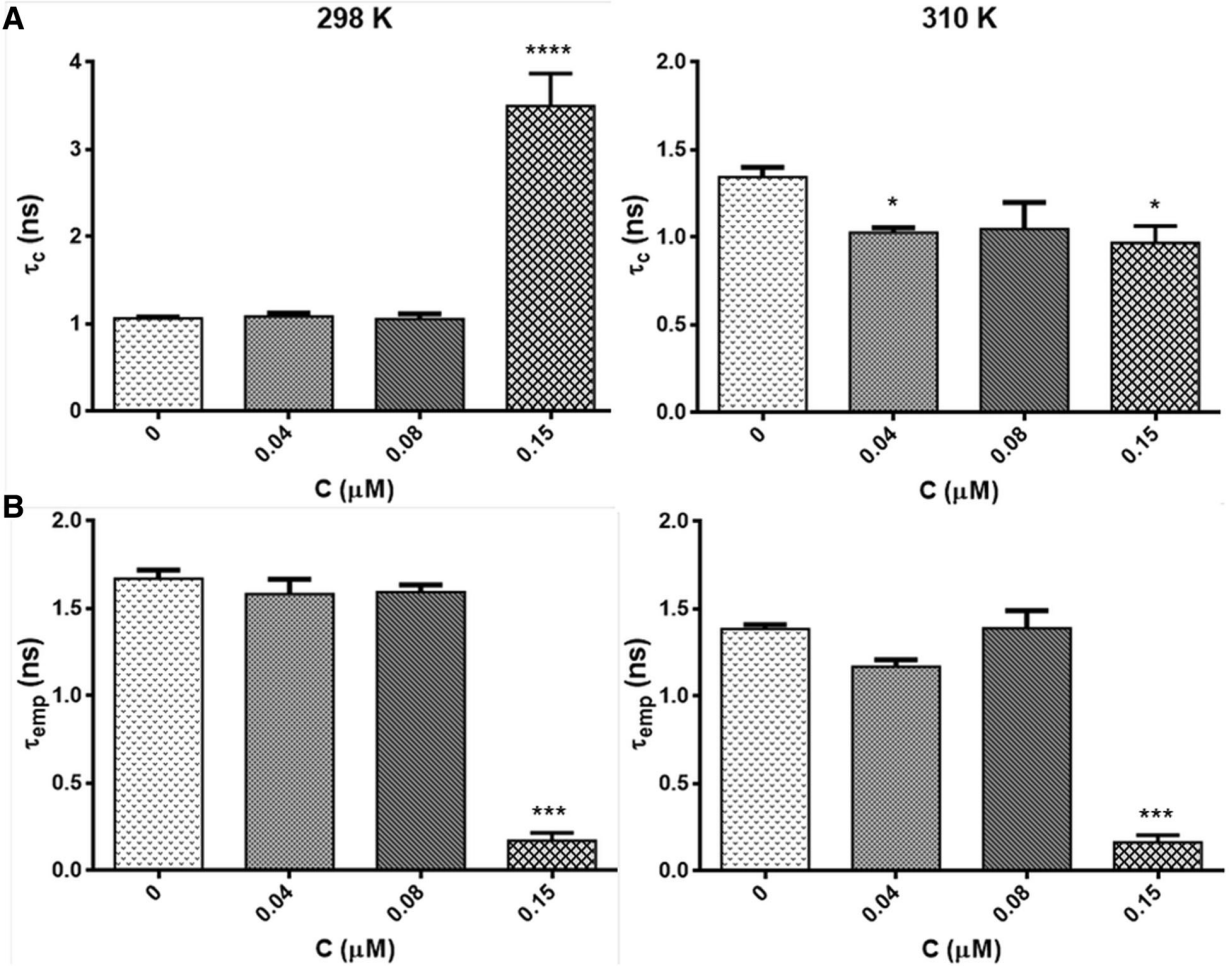
Concentration dependent change in the rotational correlation time (τ_c_) of spin label in HT-29 colon cancer cells at 298 and 310 K: **a**) from computer simulation, **b**) calculated from direct evaluation of spectra. The degree of significance was denoted as follows: *p < 0.05, **p < 0.01, ***p < 0.001 and ****p < 0.0001 with respect to control sample

To understand the effect of the HYP-mediated PDT on the dynamic properties of tumor cells, both regional and empirical correlation times were determined depending on HYP concentration. The changes in the obtained rotational correlation times from simulation program and the empirical correlation times determined from the spectra were represented in Fig. 5. From simulation results, an increase in the rotational correlation time with 0.15 μM HYP treatment was observed at 298 K, indicating restricted motion of spin label as a result of HYP association. On the contrary, a slight decrease in the rotational correlation time with HYP treatment, monitored at 310 K (Fig. 5a). Both results were obtained for the most populated domain.

As seen from Fig. 5b, the calculated correlation time, τ_emp_ decreased dramatically with 0.15 μM HYP treatment at both temperatures (Fig. 5b). τ_emp_ gives the correlation time of the observed experimental spectrum and includes the contribution of all domains within the sample. While τ_c_ gives information about the domains, τ_emp_ can be regarded as the mean correlation time for the sample. However, this was not the case at 0.15 μM HYP. Only one of the domains includes higher contribution of HYP at 0.15 μM concentration and calculated empirical correlation time (τ_emp_) was low. At this HYP concentration, τ_emp_ can only be calculated from the narrow line of the isotropic part, having the lowest populated domain and could not be taken as the mean correlation time of the whole spectrum (Fig. 2). If we consider only the HYP concentrations below 0.15 μM HYP, there is no significant change with HYP concentration and the results for the mean and most populated domain are close to each other. At 0.15 μM HYP concentration, the most populated domain (higher contribution of HYP) has higher correlation time (τ_c_) (Fig. 5a). The weighted mean of τ_c_’s gives similar results. Higher τ_c_ indicates the restricted motion of spin label, which indicates less fluid environment. Consistent with our results, in a previous EPR spin labeling study with HT-29 colon cancer cells, using a different drug celecoxib, an increase in the empirical correlation time was observed which means a decrease in fluidity. However, the drug concentrations were much higher in comparison to our study [19]. In the past, there were some studies on the correlation time of the healthy and neoplastic human tissues taken immediately after surgery, using EPR spin labeling. According to their results, there was no difference either in the mobility or line shape of the healthy and malignant cells [20]. However, in another ^31^P NMR study on human colon cancer, the change in the phospholipid profiles was shown and authors suggested their method for the characterization, diagnosis and treatment of the colorectal diseases [21]. In case of a change in the phospholipid constitution, there are changes in the surface charge and in membrane fluidity. Therefore, some changes in the EPR spectra of the samples may be expected [22]. Recent studies indicate the importance of the membrane lipid replacement for various diseases including cancer, to restore the phospholipid function in cells and tissues [23, 24].

**Fig. 6:**
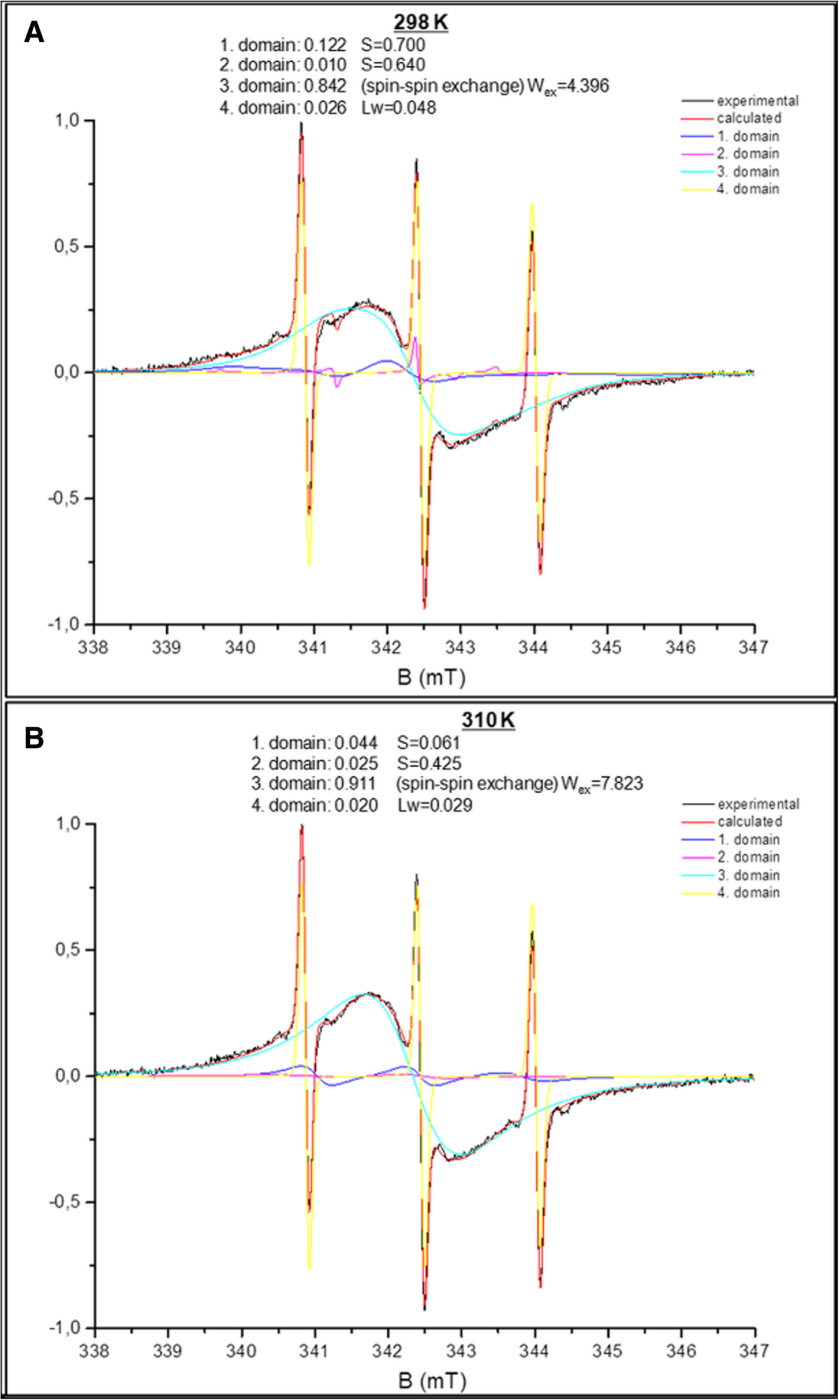
Experimental, calculated, and domain spectra of 16-DS spin labeled 0.4 μM HYP mediated HT-29 cells at **a**) 298 K and **b**) 310 K

Because of HYP accumulation, the population of more hydrophobic (less polar, pa = 0.8–0.9) environment increases in 0.15 and 0.4 μM HYP mediated samples. As spin label is also hydrophobic, the percentage of spin label, which prefers to stay in hydrophobic region, was higher. Therefore, it caused spin-spin exchange interaction, which starts at 0.15 μM HYP and more effective at 0.4 μM HYP concentration. The experimental, calculated, and domain spectra of 16-DS spin labeled 0.4 μM HYP mediated HT-29 cells at 298 and 310 K are shown in Fig. 6. 0, 0.04, 0.08 and 0.15 μM HYP mediated samples were simulated using MES, while the spectra were simulated by EPRSIM program including the spin-spin exchange contribution for 0.4 μM HYP mediated samples. It was not possible to simulate the spectra of 0.4 μM HYP mediated samples with MES because of the strong spin-spin exchange interaction.

## Conclusion

In this study, the effects of HYP-mediated PDT on structural and dynamic properties of cell membrane of the colon cancer cell line HT-29 were investigated. It is concluded that there are changes in biophysical characteristics of tumor cells caused by HYP-mediated PDT. Up to 0.15 μM concentrations of HYP, HYP-mediated PDT was not efficient on fluidity of cell membranes, but there was a slight increase in the order parameter. At 0.15 μM HYP, there was an increase in the order and weight of one domain indicating an increase in the HYP accumulation. In consistence with previous studies, the accumulation of HYP within cells by increasing concentration might be correlated with anti-carcinogenic effect of HYP-mediated PDT.

## Data Availability

All data generated or analyzed during this study are included in this published article.

## Abbreviations

16-DS: 16-doxyl stearic acid
EPR: Electron paramagnetic resonance
HT-29: Colon adenocarcinoma cells
HYP: Hypericin
MES: Anisotropic Tumbling with Partial Averaging of All Rotations model in EPRSIM-C
PDT: Photodynamic therapy
S: Order parameter
τ_c_: rotational correlation time
τ_emp_: empirical correlation time
